# Risk factors for the deterioration of periventricular–intraventricular hemorrhage in preterm infants

**DOI:** 10.1038/s41598-020-70603-z

**Published:** 2020-08-12

**Authors:** Tian Wu, Yan Wang, Tao Xiong, Sheng Huang, Tian Tian, Jun Tang, Dezhi Mu

**Affiliations:** 1 Department of Pediatrics, West China Second University Hospital, Sichuan University, Chengdu, China; 2 Key Laboratory of Birth Defects and Related Diseases of Women and Children (Sichuan University), Ministry of Education, Chengdu, China; 3 Deep Underground Space Medical Center, West China Hospital, Sichuan University, Chengdu, Sichuan China; 4 Department of Information Management, West China Second University Hospital, Sichuan University, Chengdu, Sichuan China; 5 Department of Epidemiology and Biostatistics, School of Public Health, Institute of Reproductive and Child Health, Peking University Health Science Center, Beijing, China

**Keywords:** Neonatal brain damage, Risk factors

## Abstract

Preterm infants with periventricular–intraventricular hemorrhage (PV–IVH) have a high risk of neurological sequelae, with severity depending on the severity of the PV–IVH. Previous studies on the pathogenesis of PV–IVH have focused mainly on comparisons of perinatal risk factors between patients with and without PV–IVH. Notably, most cases of PV–IVH occur within the first 3 days after birth, and the condition may worsen within 1 week following the initial diagnosis. However, the risk factors that contribute to the deterioration of PV–IVH have not been investigated. In this cohort study, 514 PV–IVH infants with a gestational age (GA) < 32 weeks were enrolled. The dependent variable was initially diagnosed as mild PV–IVH (grade I or II) that subsequently progressed to severe PV–IVH (grade III or IV) within 1 week. A stepwise forward multivariate logistic regression model was adopted to select potential or related factors that affected the deterioration of PV–IVH in preterm infants. Overall, 42 of the 514 infants with PV–IVH (8.2%) showed deterioration within 1 week. The results showed that maternal lower genital tract infection (OR 3.73, 95% CI 1.75–7.95) was an independent risk factor for PV–IVH deterioration. Higher GA (OR 0.62, 95% CI 0.48–0.80) was a protective factor. Our results suggest that maternal lower genital tract infection and a lower GA may contribute to PV–IVH deterioration in preterm infants.

## Introduction

Periventricular–intraventricular hemorrhage (PV–IVH) is a common intracranial lesion in premature infants and is characterized as bleeding into the ventricular system due to the rupture of microvessels within the germinal matrix tissue^1^. The incidence of PV–IVH is higher in neonates with a younger gestational age (GA) than in other groups and is especially high in neonates with GA < 32 weeks^2^. PV–IVH ranges in severity from grade I to grade IV according to the *Papile* grading system^3^. In the majority of infants with mild PV–IVH (grade I and II), the clinical prognosis is generally favorable^4^. However, up to approximately 60% of infants with severe PV–IVH (grades III and IV) develop cerebral palsy and/or mental retardation^5^. These severe sequelae create enormous social and financial burdens.

To date, no treatment has been able to reduce the sequelae of IVH in preterm infants^6^. Notably, PV–IVH mainly occurs within the first 3 days after birth and can progress rapidly early in life (within 1 week)^5,7^. Therefore, it is very important to detect risk factors for PV–IVH deterioration as this could help pediatricians prevent deterioration from mild to severe PV–IVH. Previous studies have evaluated the risk factors for PV–IVH in preterm infants by comparing PV–IVH infants to those without PV–IVH, and these studies have identified vaginal delivery, intrauterine infection, asphyxia, postpartum mechanical ventilation, coagulopathy, thrombocytopenia, etc. as perinatal risk factors^8,9^. Whether these risk factors contribute to PV–IVH deterioration remains unknown. Therefore, the present study was performed to explore the factors that could potentially contribute to the deterioration of PV–IVH in preterm infants with GA < 32 weeks.

## Methods

### Study population

West China Second University Hospital is regarded as an important tertiary hospital in Southwest China at which a large number of critically ill newborns are treated in the neonatal intensive care unit (NICU). Our unit has 80 beds, including 30 NICU cots and 50 cots in a general ward. Approximately 4,000 neonates with internal (medical) or surgical diseases (including cardiac surgery diseases) are admitted to our facility each year. We performed a cohort study to analyze collected data regarding preterm infants with a confirmed diagnosis of PV–IVH who were admitted to our NICU between June 2009 and March 2018. In accordance with our center’s protocols, all infants underwent a series of cranial ultrasound screens for PV–IVH per the American Academy of Neurology (AAN) practice parameters^10^. In addition to AAN practices, we also followed the Chinese practice guidelines for PV–IVH screening^11^. The Chinese practice guidelines recommended that PV–IVH screening should be performed in all preterm infants with risk factors. Therefore, almost all premature infants in our hospital are screened by cranial ultrasound.

### Diagnosis of periventricular–intraventricular hemorrhage

An initial screen was performed routinely within 72 h after birth. In cases where no PV–IVH was detected, the screen was repeated at 7, 14, and 42 days of life. If PV–IVH was identified, serial ultrasound screenings were repeated at weekly intervals until the PV–IVH was demonstrated to be stable or resolved on at least two consecutive scans. These ultrasound examinations were supervised and reported by pediatric radiologists who were blinded to the clinical details of the infants. Those pediatric radiologists had expertise and received uniform PV–IVH screening training in ultrasonography to evaluate interobserver reliability. The *Papile* grading system^3^ was used for the assessment of PV–IVH. The definitions were as follows: grade I, hemorrhage restricted to the periventricular germinal matrix regions or germinal matrix; grade II, intraventricular hemorrhage without ventricular dilatation; grade III, extended hemorrhage into dilated ventricles; and grade IV, hemorrhage within the ventricular system and parenchyma.

PV–IVH deterioration was defined as progression from mild PV–IVH to severe PV–IVH within the first week after birth. Infants with PV–IVH deterioration were included in the case group. Infants without PV–IVH deterioration served as the control group. Infants with PV–IVH deterioration were further divided into two subgroups: those with mild deterioration [grade (I or II) to grade III] and those with severe deterioration [grade (I or II) to grade IV].

### Data collection

The present research represents a retrospective study. Data were extracted from the hospital’s patient database. The study protocol was approved by the medical ethics committee of West China Second University Hospital, and written informed consent was obtained from the parents of the infants. We selected the possible risk factors according to variables reported in previous studies that explored PV–IVH risk factors as well as studies that have speculated on the involved pathophysiological mechanisms. All methods were performed in accordance with the relevant guidelines and regulations.

The following maternal and neonatal baseline characteristics were collected: GA, birth weight (BW), sex, history of fetal abnormalities (including premature birth, teras, hydatidiform mole, etc. ), parity, gravidity, multiple gestations, vaginal delivery, in vitro fertilization (IVF), gestational hypertension, intrauterine infection (chorioamnionitis or umbiliculitis), premature rupture of membranes (PROM, rupture of membranes before the onset of labor^12^), placental abnormality (including placenta previa, placental abruption, etc. ), intrahepatic cholestasis of pregnancy (ICP), anemia (blood hemoglobin < 100 g/L), amniotic fluid contamination (also known as meconium-stained amniotic fluid), lower genital tract infection (vaginal and cervical secretions were culture-positive), abnormal fetal position, and fetal intrauterine distress.

Neonatal complications included asphyxia (umbilical artery pH < 7.0^13^), pneumonia, respiratory distress syndrome (RDS), apnea (premature infants with respiratory arrest for more than 20 s and full-term infants with respiratory arrest for more than 15 s^11^), patent ductus arteriosus (PDA), scleredema, anemia (venous hemoglobin < 130 g/L or peripheral blood hemoglobin < 145 g/L^11^), and sepsis.

The reviewed laboratory tests included white blood cell (WBC) count > 25 × 10^9^ cells/L, C-reactive protein (CRP) > 8 mg/L, progressive decrease in platelets, abnormal coagulation (activated partial thromboplastin time > 70 s), blood glucose < 2.2 mmol/L, and albumin < 30 g/L. The indicators tested before or within 24 h of the initial PV–IVH diagnosis were considered the baseline values. These tests were repeated at least once every 3–5 days, and additional measurements were conducted based on infant condition. A progressive decrease in platelets was defined as a platelet count that decreased to lower than baseline value and lower than 100 × 10^9^ cells/L.

The recorded treatment protocols included invasive respiratory support (using conventional ventilation or high-frequency oscillatory ventilation after endotracheal intubation), noninvasive respiratory support (using bilevel positive airway pressure, continuous positive airway pressure, or nasal intermittent positive pressure ventilation without endotracheal intubation), plasma transfusion, and administration of pulmonary surfactant, dopamine, antibiotics, and intravenous immunoglobulin. These treatments are generally initiated within 72 h after birth.

### Statistical analysis

Statistical analyses were performed using SPSS version 19 (SPSS, Inc., Chicago, IL, USA). Chi-square tests or Fisher’s exact test were used to compare categorical variables. An independent sample *t* test was used for continuous data. Continuous variables are described as the mean and standard deviation. A stepwise method was performed to screen variables with statistical significance in the univariate analysis. Factors finally selected were used as confounding factors to calculate the adjusted odds ratios (aORs) and the corresponding 95% confidence intervals (CIs). All tests of hypotheses were 2-tailed. Statistical significance was defined as a P value < 0.05.

## Results

**Figure 1:**
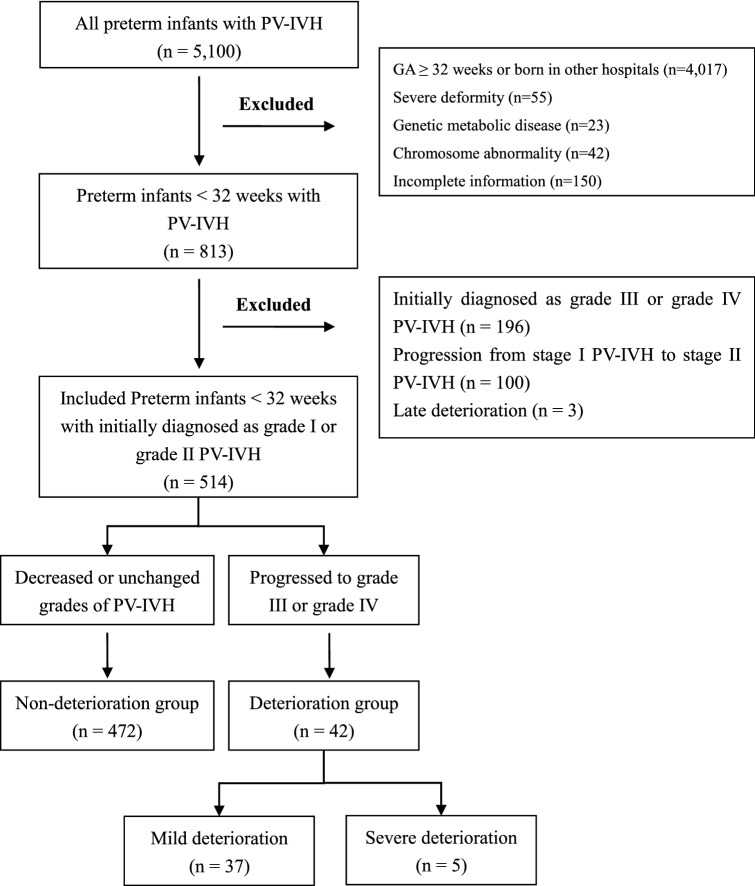
Flow chart in selection of preterm infants with PV–IVH. *Initial diagnosis of PV–IVH was within 72 h after birth. Reexamination of ultrasound was periodic after initial diagnosis.

In all, 3,783 preterm infants were diagnosed with PV–IVH during the study period. The rates of PV–IVH in different GA groups are shown in Supplementary Table 1. We included preterm infants born at less than 32 weeks GA as the target population^14^. Of the 3,783 preterm infants with PV–IVH, the 2,700 infants born at a GA of more than 32 weeks or born in other hospitals were excluded. Infants with severe deformity (n = 55), genetic metabolic diseases (n = 23), chromosome abnormalities (n = 42) or incomplete information (n = 150) were also excluded. Of the 813 remaining cases, after infants with initially severe PV–IVH (grade III or IV, n = 196) and infants with PV–IVH that progressed from grade I to grade II (n = 100) were excluded, 517 infants who were initially diagnosed with grade I or II PV–IVH remained. Because the mechanisms and causes of early deterioration (within the first week) and late deterioration (after the first week) are likely to be different^15^, 3 cases with late deterioration were excluded (Fig. 1).

A total of 514 preterm infants (GA < 32 weeks) with initially mild PV–IVH met the inclusion criteria. The mean estimated GA of these infants was 29.5 ± 1.5 weeks, and the mean BW was 1,341.0 ± 283.1 g. GA and BW met the assumptions of normality. The sex distribution was 269 (52.3%) male and 245 (47.7%) female. Overall, 42 of the 514 infants (8.2%) suffered PV–IVH deterioration within the first week after birth. The rates of different GA groups were as follows: infants with a GA < 28 weeks, 2.4% (12/495); infants with a GA 28–30 weeks, 1.6% (25/1,558); and infants with a GA 30–32 weeks, 0.12% (5/4,044) (Supplementary Table 1). Among these patients, there were 37 with mild deterioration and 5 with severe deterioration.

The demographic and antenatal features of preterm infants with PV–IVH are summarized in Table 1. We found that the rates of intrauterine infection and lower genital tract infection were significantly higher, whereas GA, BW and the rate of primiparity were significantly lower in infants with PV–IVH deterioration than in those without PV–IVH deterioration. There were no significant differences between the two groups in the rates of male sex, history of fetal abnormalities, primigravity, multiple gestations, vaginal delivery, IVF, gestational hypertension, PROM, placental abnormality, ICP, anemia, amniotic fluid contamination, abnormal fetal position, or fetal intrauterine distress.

**Table 1:** Demographic features of preterm infants with PV–IVH.

Variables	Deterioration of PV–IVH n = 42	No deterioration of PV–IVH n = 472	X^2^	P
Gestational age (weeks, mean ± SD)	28.2 ± 1.3	29.7 ± 1.4	t = 6.507	0.00
Birth weight (g, mean ± SD)	1,114.0 ± 234.6	1,361.2 ± 278.4	t = 5.579	0.00
Male (n, %)	21 (50.0)	248 (52.5)	0.100	0.75
History of fetal abnormalities (n, %)	7 (16.7)	97 (20.6)	0.361	0.55
Primigravidity (n, %)	13 (31.0)	143 (30.3)	0.008	0.93
Primiparity (n, %)	18 (42.9)	263 (55.7)	7.007	0.03
Multiple gestations (n, %)	19 (45.2)	154 (32.6)	2.747	0.10
Vaginal delivery (n, %)	29 (69.0)	293 (62.1)	0.801	0.37
In vitro fertilization (n, %)	2 (4.8)	29 (6.1)	0.130	0.72
Gestational hypertension (n, %)	5 (11.9)	21 (4.4)	*N*	0.05
Intrauterine infection (n, %)	15 (35.7)	105 (22.2)	3.909	0.04
Premature rupture of membranes (n, %)	8 (19.0)	158 (33.5)	3.671	0.06
Placental abnormality (n, %)	11 (26.2)	125 (26.5)	0.002	0.97
Intrahepatic cholestasis of pregnancy (n, %)	9 (21.4)	72 (15.3)	1.108	0.29
Anemia (n, %)	15 (35.7)	128 (27.1)	1.419	0.23
Lower genital tract infections (n, %)	30 (71.4)	186 (39.4)	16.232	0.00
Amniotic fluid contamination (n, %)	7 (16.7)	53 (11.2)	*N*	0.31
Abnormal fetal position (n, %)	0 (0.0)	9 (1.9)	0.815	0.37
Fetal intrauterine distress (n, %)	8 (19.0)	55 (11.7)	1.961	0.16

*PV–IVH* periventricular–intraventricular hemorrhage.

Table 2 shows the differences in the incidences of neonatal complications between the two groups. Only the rate of asphyxia was higher in infants with PV–IVH deterioration than in those without deterioration. There were no differences in pneumonia, RDS, apnea, PDA, scleredema, anemia or sepsis.

**Table 2:** Complications of preterm infants with PV–IVH who deteriorated or not.

Variables	Deterioration of PV–IVH n = 42	No deterioration of PV–IVH n = 472	X^2^	P
Asphyxia (n, %)	25 (59.5)	169 (35.8)	9.234	0.00
Pneumonia (n, %)	35 (83.3)	374 (79.2)	0.398	0.53
Respiratory distress syndrome (n, %)	25 (59.5)	218 (46.2)	2.752	0.10
Apnea (n, %)	23 (54.8)	295 (62.5)	0.979	0.32
Patent ductus arteriosus (n, %)	10 (23.8)	128 (27.1)	0.215	0.64
Scleredema (n, %)	7 (16.7)	42 (8.9)	*N*	0.10
Anemia (n, %)	22 (52.4)	183 (38.8)	2.979	0.08
Sepsis (n, %)	6 (14.3)	41 (8.7)	*N*	0.26

*PV–IVH* periventricular–intraventricular hemorrhage.

The comparisons of laboratory test results and treatment of the two groups are shown in Table 3. We found that WBC count > 25 × 10^9^ cells/L, CRP > 8 mg/L, invasive respiratory support, and pulmonary surfactant, dopamine, and antibiotic administration were significantly associated with PV–IVH deterioration.

**Table 3:** Laboratory test and treatment in preterm infants with PV–IVH who deteriorated or not.

Variables	Deterioration of PV–IVH n = 42	No deterioration of PV–IVH n = 472	X^2^	P
WBC > 25 × 10^9^/L^a^ (n, %)	15 (35.7)	95 (20.1)	5.571	0.02
CRP > 8 mg/L (n, %)	14 (33.3)	86 (18.2)	5.622	0.02
Progressive decrease in platelets (n, %)	4 (9.5)	27 (5.7)	*N*	0.31
Abnormal coagulation (n, %)	7 (16.7)	142 (30.1)	3.373	0.07
Blood glucose < 2.2 mmol/L (n, %)	1 (2.4)	48 (10.2)	2.713	0.10
Albumin < 30 g/L (n, %)	15 (35.7)	209 (44.3)	1.151	0.28
Invasive respiratory support (n, %)	26 (61.9)	149 (31.6)	15.807	0.00
Non-invasive respiratory support (n, %)	2 (4.8)	22 (4.7)	*N*	1.00
Pulmonary surfactant (n, %)	29 (69.0)	240 (50.8)	5.121	0.02
Dopamine (n, %)	22 (52.4)	108 (22.9)	17.763	0.00
Antibiotics (n, %)	11 (26.2)	264 (55.9)	13.714	0.00
Plasma transfusion (n, %)	2 (4.8)	14 (3.0)	*N*	0.38
Intravenous immunoglobulin (n, %)	2 (4.8)	52 (11.0)	1.605	0.21

*PV–IVH* periventricular–intraventricular hemorrhage, *WBC* white blood cell, *CRP* C-reactive protein.

^a^Baseline values were tested before or within PV–IVH diagnosis.

There were 12 variables with statistical significance in the univariate analysis. First, WBC and CRP levels were indicators of laboratory tests and thus could not be included into the multivariate analysis as influencing factors. Second, according to the results of the correlation analysis, some variables showed certain correlations with other independent variables (Supplementary Table 2). Therefore, we used a stepwise method to screen variables. As a result, GA, BW and maternal lower genital tract infection were included in the regression analysis (Supplementary Table 3). After multivariable adjustments, maternal lower genital tract infection (OR 3.73, 95% CI 1.75–7.95) was independently associated with PV–IVH deterioration, whereas greater GA (OR 0.62, 95% CI 0.48–0.80) was an independent protective factor against PV–IVH deterioration (Table 4). No statistical relation was observed between BW and PV–IVH deterioration (P = 0.05, OR 0.99, 95% CI 0.99–1.00). As Supplementary Table 4 shows, the three variables had relatively small VIFs, indicating low collinearity among the variables.

**Table 4:** Correlative factors for PV–IVH Deterioration in Preterm infant.

	β	SE	Wals	P	aOR	95% CI
Gestational age (weeks)	− 0.483	0.134	12.948	0.00	0.62	0.48–0.80
Birth weight (kilogram)	− 0.002	0.001	4.296	0.05	0.99	0.99–1.00
Lower genital tract infection	1.315	0.387	11.566	0.00	3.73	1.75–7.95

Adjusted for: gestational age, birth weight, lower genital tract infection.

*PV–IVH* periventricular–intraventricular hemorrhage, *aOR* adjusted odds ratio, *95% CI* 95% confidence interval.

The mean GA was significantly lower in the mild deterioration group (28.3 ± 1.6 weeks) than in the severe deterioration group (29.7 ± 1.6 weeks) (Supplementary Table 5). The rates of WBC count > 25 × 10^9^/L and CRP > 8 mg/L were higher in infants in the severe deterioration group than in those in the mild deterioration group (Supplementary Table 6). There were no differences in other factors and complications of preterm infants (Supplementary Table 7).

## Discussion

To the best of our knowledge, this is the first study to assess the risk factors that contribute to the early deterioration of PV–IVH, which have previously been poorly explored. Our study results indicate that 8.2% of infants with mild PV–IVH suffer from deterioration of PV–IVH within the first week. Maternal lower genital tract infection and lower GA were risk factors for PV–IVH deterioration. Notably, maternal lower genital tract infections may induce preterm birth (shorter GA), indicating the importance of infection for PV–IVH deterioration.

We found that the risk of PV–IVH deterioration increased as GA decreased. As several previous reports have shown, a shorter GA is related to a higher risk of severe PV–IVH^16–18^. We speculated that several mechanisms may be involved in this relationship, as follows. The deterioration in PV–IVH observed in infants with shorter GA is associated with the poor coagulation due to immature liver function^16,19^. The microvasculature of the germinal matrix in the subependymal layer is very fragile in very preterm infants due to the incomplete development of the endometrial microvasculature and a lack of collagen IV^20^. Autoregulatory mechanisms governing cerebral blood flow are also poorly developed in the immature brain. Therefore, when preterm infants have hypoxia, hypercapnia, and acidosis, the cerebral blood flow velocity fluctuates, and microvessels lack protection by the basement membrane and are more susceptible to rupture^21,22^. In addition, lower GA was generally associated with more preterm complications and treatments. In our study, invasive mechanical ventilation, PS, and dopamine administration were more commonly used in infants with PV–IVH deterioration. This observation could be explained by the lower GA in infants with PV–IVH deterioration than in infants without deterioration. For the above reasons, when PV–IVH occurs in infants with shorter GA, they are more prone to deterioration than was found for infants with a longer GA. Luque et al.^7^ developed a risk prediction model for severe IVH in infants with a BW less than 1,500*g*. They found that GA was the major contributor to the model. Some studies have also indicated that GA reflects fetal maturity more accurately than BW does among preterm infants^23–25^. Similar to their findings, we found that GA rather than BW had a impact on PV–IVH progression. Therefore, the prevention of preterm birth is an important way to prevent the development of PV–IVH.

As many previous studies have reported, infections and inflammatory responses are strongly associated with neonatal brain injury (including IVH, periventricular leukomalacia and hypoxic ischemic encephalopathy)^26–28^. In our study, maternal lower genital tract infections increased the risk of PV–IVH deterioration more than three-fold. According to a meta-analysis by Huang et al.^29^, antenatal infection, including chorioamnionitis, funisitis and sepsis, may increase the risk of the development of PV–IVH in preterm infants. Maternal lower reproductive tract infection is one of the important transmission methods of intrauterine infection^30^. In this study, not only the ratio of maternal lower reproductive tract infection but also the ratio of elevated WBC count and CRP level were increased in infants with PV–IVH deterioration. Recent clinical and experimental data support the view that cytokines released during intrauterine infection are a direct cause of injury to the immature brain^31^. Elevated cytokine levels in preterm infants were associated with an increased risk of PV–IVH^32^. Different hypotheses have been proposed to explain the underlying mechanisms. First, inflammatory factors may promote the deterioration of PV–IVH by increasing cerebral oxygen consumption, breaking brain barriers, and activating the immune response in preterm infants^29^. Second, the autoregulatory function of cerebral blood pressure is immature in preterm infants. Infection may induce abnormal fluctuations in blood pressure, resulting in unstable cerebral blood pressure, and thereby contributing to the development of PV–IVH^33,34^. Therefore, early identification and management of antenatal infection is important to prevent IVH deterioration.

Generally, the severity of illness observed in infants who exhibited IVH deterioration was consistent with that observed in infants without deterioration. No difference was observed between the groups in complications, including pneumonia, RDS, apnea, PDA, scleredema, anemia and sepsis. Furthermore, asphyxia was excluded from the final model by the stepwise method in the regression analysis, although the rate of asphyxia was different between the two groups in the univariate analysis. Previous studies on the relationship between asphyxia and PV–IVH have produced conflicting results^35,36^. Xu et al.^35^ reported that asphyxia was an important risk factor for PV–IVH (OR = 5.65) in premature infants with mechanical ventilation. Konstantinidis et al.^36^ also found that asphyxia contributes to the development of PV–IVH and hypoxic-ischemic encephalopathy. However, van Iersel et al.^37^ found that perinatal asphyxia in preterm infants was not an additional risk factor for PV–IVH. Some explanations may explain this inconsistency. First, it is conceivable that preterm infants have high tolerance to asphyxia, potentially due to the high plasticity of the preterm brain. Second, the effect of asphyxia on PV–IVH deterioration may be overcome by the effects of other risk factors, such as perinatal infection^37^. Asphyxia was defined as an umbilical artery pH < 7.0 in our study, and this may also be a clinical manifestation of antenatal infection. Third, the small sample size and lack of control for unknown risk factors may be one cause of the disagreement. Therefore, the effect of asphyxia on PV–IVH deterioration in premature infants remains to be further studied.

There are some limitations to our study. First, our study started with a very large cohort but ultimately included only a small number of babies < 28 weeks with PV–IVH. Because this was a single-center study of preterm infants (GA < 32 weeks) born in China, our results should be generalized with caution in other settings. Second, our data may suffer from bias due to the retrospective nature of this study. Third, there may have been detection bias between radiologists (interobserver reliability). To evaluate interobserver reliability, we excluded the most confusing population according to radiologists (progression from PV–IVH grade I to grade II) from consideration in this study. Fourth, the definitions used for some factors in our investigation may not be consistent with those used in other studies. Nevertheless, in this study, we firstly identify the risk factors for early PV–IVH deterioration in a Chinese NICU by using strict PV–IVH screening guidelines and carefully considered risk factors. This investigation should serve as a basis for future prospective multicenter trials.

## Conclusions

In conclusion, maternal lower genital tract infection and lower GA may be risk factors for early PV–IVH deterioration in preterm newborns with GA < 32 weeks. In view of these findings, it is important to prevent maternal lower genital tract infections or diagnose them early.

## Supplementary information

Supplementary Tables.
