# Supplementary material for: Risk factors for the deterioration of periventricular–intraventricular hemorrhage in preterm infants
Source: Sci Rep. 2020 Aug 12;10:13609. doi: 10.1038/s41598-020-70603-z (PMC7423930; doi:10.1038/s41598-020-70603-z)
Supplement: Supplementary file 1 — Supplementary Tables. [file 41598_2020_70603_MOESM1_ESM.pdf]

Risk factors for the deterioration of periventricular-intraventricular hemorrhage in preterm infants

Tian Wu, MD[a,b], Yan Wang, MD[a,b], Tao Xiong, MD, PhD[a,b,c,*], Sheng Huang, MSE[d], Tian Tian MS [e], Jun Tang, MD, PhD [a,b], Dezhi Mu, MD, PhD [a,b]

[a] *Department of Pediatrics, West China Second University Hospital, Sichuan University, Chengdu, China*

[b] *Key Laboratory of Birth Defects and Related Diseases of Women and Children (Sichuan University), Ministry of Education, Chengdu, China*

[c] *Deep Underground Space Medical Center, West China Hospital, Sichuan University, Chengdu, Sichuan, China*

[d] *Department of information management, West China Second University Hospital, Sichuan University, Chengdu, Sichuan, China*

[e] *Institute of Reproductive and Child Health, Department of Epidemiology & Biostatistics, School of Public Health, Peking University Health Science Center, Beijing, China*

TW and YW contributed equally to this work.

*Correspondence: Tao Xiong, No. 20, Section Three, South Renmin Road, Chengdu, Sichuan 160041, China (e-mail: tao_xiong@126.com).

The authors report no conflicts of interest.

Supplementary Table 1. The PV-IVH deterioration in different gestational groups [%(n)].

	**< 28 weeks**<br>**n=495**	**28~30 weeks**<br>**n=1,558**	**30~32 weeks**<br>**n=4,044**	**32~37 weeks**<br>**n=18,179**
PV-IVH, %(n)	55.6 (275)	40.1 (625)	28.1 (1,133)	9.6 (1,750)
PV-IVH deterioration, %(n)	2.4 (12)	1.6 (25)	0.19 (8)	N
PV-IVH deterioration within first week, %(n)	2.4 (12)	1.6 (25)	0.12 (5)	N

PV-IVH: periventricular - intraventricular hemorrhage

Supplementary Table 2. Correlation analysis.

		GA	BW	Intrauterine infection	Asphyxia	Primiparity	Invasive respiratory support	PS	Dopamine	Lower genital tract infection	Antibiotics
GA	r	1.000	0.625**	-0.152**	-0.196**	0.013	-0.363**	-0.156**	-0.229**	-0.166**	0.134**
	p		0.000	0.001	0.000	0.769	0.000	0.000	0.000	0.000	0.002
BW	r	0.625**	1.000	-0.197**	-0.158**	0.066	-0.488**	-0.187**	-0.366**	-0.303**	0.171**
	p	0.000		0.000	0.000	0.132	0.000	0.000	0.000	0.000	0.000
Intrauterine infection	r	-0.152**	-0.197**	1.000	-0.022	-0.015	0.196**	0.122**	0.197**	0.434**	-0.261**
	p	0.001	0.000		0.623	0.738	0.000	0.006	0.000	0.000	0.000
Asphyxia	r	-0.196**	-0.158**	-0.022	1.000	-0.073	0.076	0.020	0.175**	-0.012	-0.044
	p	0.000	0.000	0.623		0.098	0.086	0.653	0.000	0.779	0.319
Primiparity	r	0.013	0.066	-0.015	-0.073	1.000	-0.096*	-0.032	-0.082	-0.096*	0.075
	p	0.769	0.132	0.738	0.098		0.029	0.472	0.065	0.030	0.088
Invasive respiratory support	r	-0.363**	-0.488**	0.196**	0.076	-0.096*	1.000	0.110*	0.300**	0.353**	-0.200**
	p	0.000	0.000	0.000	0.086	0.029		0.012	0.000	0.000	0.000

PS	r	-0.156**	-0.187**	0.122**	0.020	-0.032	0.110*	1.000	0.295**	0.244**	-0.099*
	p	0.000	0.000	0.006	0.653	0.472	0.012		0.000	0.000	0.025
Dopamine	r	-0.229**	-0.366**	0.197**	0.175**	-0.082	0.300**	0.295**	1.000	0.511**	-0.304**
	p	0.000	0.000	0.000	0.000	0.065	0.000	0.000		0.000	0.000
Lower genital	r	-0.166**	-0.303**	0.434**	-0.012	-0.096*	0.353**	0.244**	0.511**	1.000	-0.506**
tract infection	p	0.000	0.000	0.000	0.779	0.030	0.000	0.000	0.000		0.000
	r	0.134**	0.171**	-0.261**	-0.044	0.075	-0.200**	-0.099*	-0.304**	-0.506**	1.000
Antibiotics	p	0.002	0.000	0.000	0.319	0.088	0.000	0.025	0.000	0.000	

***** P < 0.05 between the two variables.

** P < 0.01 between the two variables.

GA: gestational age; BW: birth weight; PS: pulmonary surfactant.

Supplementary Table 3. Stepwise regression.

	Variables	β	SE	Wals	P	OR	95%CI	
Step 1	BW	-0.001	0.001	2.125	0.14	1.00	1.00	1.00
	Intrauterine infection	-0.124	0.398	0.098	0.75	0.88	0.41	1.93
	Asphyxia	0.567	0.375	2.293	0.13	1.76	0.85	3.68
	Primarity	-0.290	0.358	0.655	0.42	0.75	0.37	1.51
	Invasive respiratory support	0.183	0.397	0.213	0.64	1.20	0.55	2.62
	PS	0.366	0.394	0.863	0.35	1.44	0.67	3.12
	Dopamine	0.053	0.439	0.015	0.90	1.05	0.45	2.49
	Antibiotics	-0.470	0.728	0.416	0.52	0.63	0.15	2.60
	Lower genital tract infection	0.776	0.743	1.091	0.30	2.17	0.51	9.33
	GA	-0.467	0.139	11.343	0.00	0.63	0.48	0.82
Step 2	BW	-0.001	0.001	2.178	0.14	1.00	1.00	1.00
	Asphyxia	-0.129	0.396	0.106	0.74	0.88	0.40	1.91
	Primarity	0.579	0.363	2.541	0.11	1.78	0.88	3.64
	Invasive respiratory support	-0.290	0.358	0.658	0.42	0.75	0.37	1.51
	PS	0.185	0.397	0.217	0.64	1.20	0.55	2.62

	Dopamine	0.374	0.388	0.932	0.33	1.45	0.68	3.11
	Antibiotics	-0.475	0.724	0.431	0.51	0.62	0.15	2.57
	Lower genital tract infection	0.802	0.710	1.278	0.26	2.23	0.56	8.96
	GA	-0.468	0.139	11.388	0.00	0.63	0.48	0.82
Step 3	BW	-0.001	0.001	2.197	0.14	1.00	1.00	1.00
	Asphyxia	0.585	0.363	2.606	0.11	1.80	0.88	3.66
	Primarity	-0.302	0.356	0.718	0.40	0.74	0.37	1.49
	Invasive respiratory support	0.184	0.396	0.216	0.64	1.20	0.55	2.62
	PS	0.373	0.387	0.926	0.34	1.45	0.68	3.10
	Antibiotics	-0.456	0.728	0.393	0.53	0.63	0.15	2.64
	Lower genital tract infection	0.766	0.707	1.175	0.28	2.15	0.54	8.61
	GA	-0.464	0.138	11.370	0.00	0.63	0.48	0.82
Step 4	BW	-0.001	0.001	2.788	0.09	1.00	1.00	1.00
	Asphyxia	0.584	0.363	2.590	0.11	1.79	0.88	3.65
	Primarity	-0.307	0.356	0.746	0.39	0.74	0.37	1.48
	Invasive respiratory support	0.347	0.383	0.822	0.36	1.42	0.67	3.00
	PS	-0.482	0.721	0.446	0.50	0.62	0.15	2.54

	Lower genital tract infection	0.810	0.697	1.349	0.25	2.25	0.57	8.81
	GA	-0.467	0.137	11.590	0.00	0.63	0.48	0.82
Step 5	BW	-0.002	0.001	2.910	0.09	1.00	1.00	1.00
	Asphyxia	0.591	0.362	2.661	0.10	1.81	0.89	3.67
	Primarity	-0.345	0.351	0.964	0.33	0.71	0.36	1.41
	PS	0.352	0.383	0.845	0.36	1.42	0.67	3.01
	Lower genital tract infection	1.201	0.394	9.318	0.00	3.33	1.54	7.19
	GA	-0.459	0.136	11.326	0.00	0.63	0.48	0.83
Step 6	BW	-0.002	0.001	3.068	0.08	1.00	1.00	1.00
	Asphyxia	0.546	0.360	2.302	0.13	1.73	0.85	3.49
	Primarity	-0.342	0.351	0.947	0.33	0.71	0.36	1.41
	Lower genital tract infection	1.262	0.388	10.585	0.00	3.53	1.65	7.56
	GA	-0.463	0.137	11.410	0.00	0.63	0.48	0.82
Step 7	BW	-0.002	0.001	3.162	0.08	1.00	1.00	1.00
	Asphyxia	0.545	0.360	2.297	0.13	1.72	0.85	3.49
	Lower genital tract infection	1.300	0.386	11.337	0.00	3.67	1.72	7.82
	GA	-0.465	0.136	11.623	0.00	0.63	0.48	0.82

Step 8	BW	-0.002	0.001	4.296	0.05	0.99	0.99	1.00
	Lower genital tract infection	1.315	0.387	11.566	0.00	3.73	1.75	7.95
	GA	-0.483	0.134	12.948	0.00	0.62	0.48	0.80

GA: gestational age; BW: birth weight; PS: Pulmonary surfactant.

Supplementary Table 4. Collinearity analysis – VIF.

	Standardized Coefficients			Collinearity Statistics	
**Model**	**β**	**t**	**P**	**Tolerance**	**VIF**
Constant	--	5.324	0.00	--	--
Birth weight	-0.067	-1.225	0.22	0.59	1.70
Gestational age	-0.221	-4.204	0.00	0.64	1.57
Lower genital tract infection	0.132	2.990	0.00	0.90	1.12

VIF less than 10 indicates no collinearity.

Supplementary Table 5. Demographic features of preterm infants with mild PV-IVH deterioration and severe PV-IVH deterioration.

Variables	Severe Deterioration n=5	Mild Deterioration n=37	P
Gestational age (weeks, mean ±SD)	29.7 ±1.6	28.3 ±1.6	**0.04**
Birth weight (gram, mean ±SD)	1256.7 ±398.9	1113.1 ±209.3	0.18
Male (n, %)	3 (60.0)	18 (48.6)	>0.99
History of fetal abnormalities (n, %)	1 (20.0)	6 (16.2)	>0.99
Primigravidity (n, %)	4 (80.0)	26 (70.2)	>0.99
Primiparity (n, %)	4 (80.0)	14 (37.8)	0.23
Multiple gestations (n, %)	0 (0)	19 (51.4)	0.05
Vaginal delivery (n, %)	3 (60.0)	26 (70.3)	0.64
In vitro fertilization (n, %)	0 (0)	2 (5.4)	>0.99
Gestational hypertension (n, %)	0 (0)	5 (13.5)	>0.99
Intrauterine infection (n, %)	2 (40.0)	13 (35.1)	>0.99
Premature rupture of membranes (n, %)	1 (20.0)	7 (18.9)	>0.99
Placental abnormality (n, %)	2 (40.0)	9 (24.3)	0.59
Intrahepatic cholestasis of pregnancy (n, %)	1 (20.0)	8 (88.9)	>0.99

Anemia (n, %)	2 (14.0)	13 (35.1)	>0.99
Lower genital tract infections (n, %)	4 (80.0)	26 (70.3)	>0.99
Amniotic fluid contamination (n, %)	1 (20.0)	6 (16.2)	>0.99
Fetal intrauterine distress (n, %)	2 (40.0)	6 (16.2)	0.24

PV-IVH: periventricular - intraventricular hemorrhage

Supplementary Table 6. Complications of preterm infants with mild PV-IVH deterioration and severe PV-IVH deterioration.

Variables	Severe Deterioration n=5	Mild Deterioration n=37	P
Asphyxia (n, %)	4 (80.0)	21 (56.8)	0.63
Pneumonia (n, %)	4 (80.0)	31 (83.8)	>0.99
Respiratory distress syndrome (n, %)	3 (60.0)	22 (59.5)	>0.99
Apnea (n, %)	3 (60.0)	16 (43.2)	0.64
Patent ductus arteriosus (n, %)	1 (20.0)	9 (24.3)	>0.99
Scleredema (n, %)	1 (20.0)	6 (16.2)	>0.99
Anemia (n, %)	2 (40.0)	20 (54.1)	0.66
Sepsis (n, %)	0 (0.0)	6 (16.2)	>0.99

PV-IVH: periventricular - intraventricular hemorrhage

Supplementary Table 7. Laboratory test and treatment in preterm infants with mild PV-IVH deterioration and severe PV-IVH deterioration.

**Variables**	**Severe Deterioration n=5**	**Mild Deterioration n=37**	**P**
WBC >25×10^9/L *(n, %)	5 (100.0)	10 (27.0)	**0.00**
CRP>8mg/L (n, %)	4 (80.0)	10 (27.0)	**0.04**
Progressive decrease in platelets (n, %)	1 (20.0)	3 (8.1)	0.41
Abnormal coagulation (n, %)	0 (00.0)	7 (18.9)	0.57
Blood glucose < 2.2mmol/L (n, %)	1 (20.0)	0 (0.0)	0.12
Albumin<30g/L (n, %)	1 (20.0)	14 (37.8)	0.64
Invasive respiratory support (n, %)	3 (60.0)	23 (62.2)	>0.99
Non-invasive respiratory support (n, %)	0 (0.0)	2 (5.4)	>0.99
Pulmonary surfactant (n, %)	3 (60.0)	26 (70.3)	>0.99
Dopamine support (n, %)	1 (20.0)	19 (51.4)	0.35
Antibiotics (n, %)	1 (20.0)	10 (27.0)	>0.99
Plasma transfusion (n, %)	0 (0.0)	2 (5.4)	>0.99
Intravenous immunoglobulin (n, %)	0 (0)	2 (5.4)	>0.99

[*]Baseline values were tested before or within 24 hours of the initial PV-IVH diagnosis. PV-IVH: periventricular - intraventricular hemorrhage; WBC: white blood cell; CRP: C-reactive protein
